# A 3-Dimensional Printed Patient-Specific Surgical Guide to Facilitate Transsphenoidal Hypophysectomy in Dogs

**DOI:** 10.3389/fvets.2022.930856

**Published:** 2022-06-20

**Authors:** Leticia Escauriaza, Joe Fenn, John McCue, Darren Roper, Helene Vandenberghe, George Nye, Bill Oxley, Nicolas Granger

**Affiliations:** ^1^Neurology Department, Bristol Veterinary Specialists at Highcroft, CVS Referrals, Bristol, United Kingdom; ^2^Department of Clinical Science and Services, Royal Veterinary College, Hertfordshire, United Kingdom; ^3^Animal Medical Centre, New York, NY, United States; ^4^Vet3D, Kendal, United Kingdom

**Keywords:** 3D printing, computed tomography, companion dogs, pituitary dependent hyperadrenocorticism, neurosurgery, hypophysectomy

## Abstract

**Objective:**

Hypophysectomy in dogs is a difficult surgery that requires specific learning and training. We aimed to evaluate the accuracy of a 3-dimensional printed patient-specific surgical guide to facilitate choosing the entry point in the basisphenoid bone before approaching the *sella turcica* during transsphenoidal hypophysectomy in dogs.

**Methods:**

Two canine cadavers and 8 dogs undergoing transsphenoidal hypophysectomy for Cushing's disease treatment, involving design and fabrication of a 3-dimensional printed guide. The ideal entry point in the basisphenoid bone outer cortical layer was determined in each dog pre-operatively; its anatomical location was described with a set of measurements then compared to post-operative computed tomography measures describing the location of the outer cortical window created in the basisphenoid bone.

**Results:**

Several guide designs were proposed, and a consensus reached based on surgeons' experience performing hypophysectomy. The device chosen could be applied to the size and shape of skulls encountered in this case series. The pre-planned measurements were comparable to post-operative measurement (there was also no statistical difference), with median of differences <0.1 mm, which we judged as clinically acceptable.

**Clinical Significance:**

Hypophysectomy in dogs is a challenging procedure that has a learning curve and needs to be performed by specialist neurosurgeons. We propose that a low-profile 3-dimensional printed surgical guide can aid the specialist neurosurgeon to locate the burring site of the outer cortical layer of the basisphenoid bone at a pre-defined location and with good accuracy. It does not alleviate the need to understand the anatomy of the region and to know how to create a slot within the basisphenoid bone, which remains essential to enter the *sella turcica*. This device could help specialist veterinary neurosurgeons wishing to be trained to perform hypophysectomy.

## Introduction

Pituitary-dependent hyperadrenocorticism is a chronic, progressive, and eventually fatal condition, present in 80–85% of dogs with naturally occurring hyperadrenocorticism (1–3). It is caused by the presence of a functional corticotroph pituitary mass secreting the adrenocorticotropic hormone (ACTH) and subsequent adrenal cortisol release (4). These tumours are usually classified histologically as adenoma, invasive adenoma, or adenocarcinoma (5). Rarely, they can secrete other hormones such as somatotropin or prolactin, or be non-functional (5, 6). Historically, pituitary masses have been classified using computed tomography (CT) or magnetic resonance imaging (MRI) by the pituitary height (mm)/brain area (mm^2^) ratio (P/B); a P/B ratio > 0.31 defines enlarged adenomas, whereas a P/B ratio < 0.31 defines non-enlarged adenomas. Enlarged adenomas can cause compression of adjacent brain structures and lead to a variety of neurological signs (7–9).

Treatment of pituitary-dependent hyperadrenocorticism can be medical or surgical with or without radiotherapy. Etiological treatment requires a transsphenoidal craniectomy (i.e., hypophysectomy) to remove the pituitary gland and associated neoplasm. Transsphenoidal hypophysectomy is becoming increasingly popular for dogs with pituitary-dependent hyperadrenocorticism, judging by recent publications, and is considered the treatment of choice for dogs with Cushing's disease (1, 9–17). According to Hanson et al. (12) looking at 150 dogs, a remission rate of 84% is seen after surgery and the 1, 2, 3, and 4-year survival rates are 83.5, 76.1, 71.5, and 67.8%, respectively (12). The same authors extended their data to 306 dogs and found the 1, 2, 3, 4, and 5-year survival rates after surgery to be 86, 79, 74, 72, and 64%, respectively (4). The mortality rate within 4 weeks of transsphenoidal hypophysectomy has been quoted to range from 8.8 to 19% (4, 14). Recently, a 5-point MRI-based grading system for pituitary masses has been proposed by Sato et al. (18), using the height and width of the pituitary tumour, extension cranio-caudally toward the optic chiasm and mamillary bodies, respectively, and extension dorsally toward the third ventricle or inter-thalamic adhesion (18). Sato et al. further classified these lesions as “type A” when there is no involvement of the surrounding arterial circle of Willis or cavernous sinus, and as “type B” when the vascular structures are involved (18). This classification is useful because dogs with grade 1 or 2 lesions can be cured (3 out of 3 grade 1A cases, 3 out of 3 grade 2A cases), grade 3 lesions may be removed fully macroscopically (22 out of 23 grade 3A cases and one grade 3B case) but recurrence can be observed (3 out of 22 grade 3A cases where the lesion had been macroscopically removed), grade 4 lesions can only be partially removed (two 4B cases were incompletely removed and one dog relapsed), and grade 5 were considered not suitable for transsphenoidal surgical treatment because only a partial resection could be expected without cure (18). In the largest case series so far published and capturing 306 dogs with Cushing's disease treated with hypophysectomy, 27 dogs died within 4 weeks and had a median P/B ratio of 0.54 (therefore above the accepted cut-off of >0.31 defining enlarged adenoma), whereas dogs that survived more than 4 weeks after surgery had a median P/B ratio of 0.38 (4, 19). In another large case series of 150 dogs from the same authors, dogs with a pituitary height >10 mm only had a survival rate of 50% in the year after surgery (12). Furthermore, in Mamelak et al. study, 4 dogs had a P/B ratio above 1, and 3 of these dogs died in the first 3 days after surgery, while one survived for 1,095 days (14).

Transsphenoidal hypophysectomy remains a challenging surgery regardless of the size of the mass because of the deep location of the *sella turcica* at the base of the skull. The surgeon needs to identify an adequate point of entry into the basisphenoid bone, while the space through the open mouth to visualise it is limited. To identify the location of the *sella turcica* and determine an entry point in the basisphenoid bone, veterinary surgeons have used palpation of the *hamuli* processes (along with other techniques discussed later), but this is challenging because of the position of the head in the surgery, usually held tilted from horizontal (9, 15, 20). The surgeon can use the inter-sphenoidal suture as an anatomical landmark, but its location varies depending on the dog's skull shape and size, or the location of the emissary vein, although, again, this vein is inconsistently present in dogs (9, 16, 21). The vascular structures (the arterial circle of Willis and cavernous venous sinus) surrounding the *sella turcica* limit the size of the window that the surgeon can create within the basisphenoid bone (1, 16). If the entry point is too far rostral, one risks entering the pre-sphenoid bone below the optic chiasm, and if the entry point is too caudal, one risks penetrating the promontory of the dorsum sellae and damage to the caudal communicating arteries (1, 4, 12, 14). Large lesions are intimately associated with the vasculature and surgical reports clearly establish occurrence of severe haemorrhage in surgery in dogs with large pituitary masses (4, 12, 14, 20).

The objective of this study was to create a low-profile (i.e., thin) and versatile system that would aid the surgeon to accurately locate the burring site and its size in the outer cortical layer of the basisphenoid bone, before continuing the approach through the basisphenoid bone and inner cortex and into the *sella turcica*. To achieve this, a 3-dimensional (3D) printed surgical guide was designed for each dog. The location and dimension of the bone window created on the outer cortex of the basisphenoid bone was compared to pre-operatively determined measures.

## Materials and Methods

### Cadaver Study

Two cadavers were donated by the pathology service at the Royal Veterinary College (London, UK) in 2018. Head dissections were performed with the use of a scalpel, periosteal elevator, and Gelpi retractors to incise the soft palate, basisphenoid mucosa and periosteum and allow visualisation of the basisphenoid bone. This was performed first to aid deciding what landmarks could easily be used to hold a surgical guide in place, and secondly, to assess whether dummy guides would permit burring of the outer basisphenoid cortical layer. Given the limited space in the oral cavity, a device occupying the least possible space was a key requirement. A CT scan of both heads was performed with the mouth held open with a 16-s line multidetector CT scanner (Siemens, Somatom Scope, Erlangen, Germany), and the following parameters were applied: pitch 0.6, rotation speed 1.5 seconds, Kvp 130, mAs static 200, slice thickness and number of slices per rotation 16 × 0.75 mm. The oral cavity was held open to avoid contact between the upper and lower arcade teeth, to allow optimal surface rendering of the teeth when reconstructing the images. The 3D printed guides for each cadaver were designed (see result section) and mock transsphenoidal hypophysectomy performed in both cadavers. Another CT scan was then taken after the mock hypophysectomy to cheque the accuracy of the burring sites.

### Construction of the 3D Printed Patient-Specific Guide

**Figure 1:**
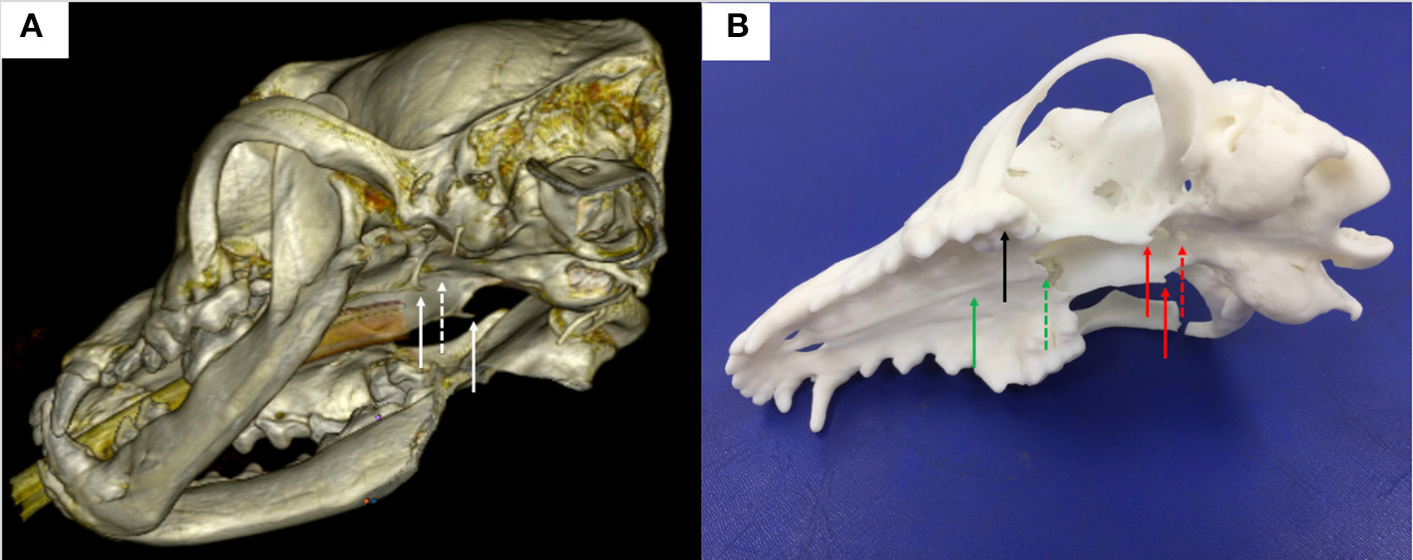
**(A)** 3-dimensional reconstruction of a canine head in a bone window using DICOM images generated from CT and the medical imaging software Horos; note the *hamuli* processes, part of the pterygoid bone (plain white arrows) and the basisphenoid bone where a window needs to be burred (dashed white arrow) in the midline between the *hamuli* processes; **(B)** 3-dimension printed skull from the DICOM images; note again the *hamuli* processes (plain red arrows) and the basisphenoid bone where a window has been burred (dashed red arrow) in the midline between the *hamuli* processes; other anatomical landmarks used for the surgical guide design were the upper teeth (black plain arrow), the hard palate (green plain arrow) and the vomer (green dashed arrow).

The burring guides were manufactured by Vet3D (Coventry, UK) using the following protocol. For each case, the CT DICOM images from a medical imaging software (Osirix, Pixmeo, SARL; Geneva, Switzerland) (Figure 1A) were exported to a computer aided design (CAD) software (Netfabb professional, Netfabb GmbH: Parsberg, Germany) and a surface rendered virtual 3D model of the skull was created, which also allowed to 3D print the skull (Figure 1B). To design a patient-specific guide, we focused on the anatomy of the upper arcade including the upper teeth, the basisphenoid bone, *hamuli* processes of the pterygoid bone, vomer, and pituitary fossa. The skull and the guide were 3D printed in methacrylate photopolymer resin with Form 3 printers using High Temperature and BioMed Amber resin, respectively (Formlabs, Somerville, Massachusetts, United States). The BioMed Amber resin used to print the guide is certified as autoclavable and biocompatible (EN ISO 10993-1:2018; 10993-3:2014; 10993-5:2009). Prior to surgery, the guides and skull model were sterilised in ethylene oxide for 24 h or in an autoclave (121°C for 20 min).

### *In vivo* Study

Eight cases were recruited between 2019 and 2022 from two institutions.

### Pituitary Imaging

The pituitary lesions and bony anatomy were assessed *via* CT in all dogs. The CT from one hospital was a 16-s line multidetector CT scanner (Siemens, Somatom Scope, Erlangen, Germany) used with the following parameters: Kvp 130, pitch 0.55, mAs modulation Care Cose 4D^TM^ turned on quality ref 220, rotation speed 1.5 s, slice thickness and number of slices per rotation 16 × 0.75 mm (with both bone and soft tissue reconstruction) and medium sharpening kernel algorithm. Dogs received an intravenous contrast injection of 1.7 ml per kg of body weight of iohexol (Omnipaque; GE Healthcare AS, Norway). The CT scan from the other hospital was a 320-s line multidetector CT scanner (Aquilion One Genesis, Canon Medical Systems). The settings used were pitch 0.625, rotation speed 0.75 s, kVp 120, slice thickness 2 mm, slice interval 1 mm, 40 slices, mA 300. The height of the lesion contained in the *sella turcica* and the area of the brain taken from the same image (thus defining the P/B ratio) were measured on transverse soft-tissue window post-contrast CT image, using the P/B cut-off of 0.31 to define non-enlarged *vs*. enlarged pituitary tumours (19). Following pituitary imaging, dogs were recovered from general anaesthetic and returned for surgery when the guide was ready.

### Surgical Technique

**Figure 2:**
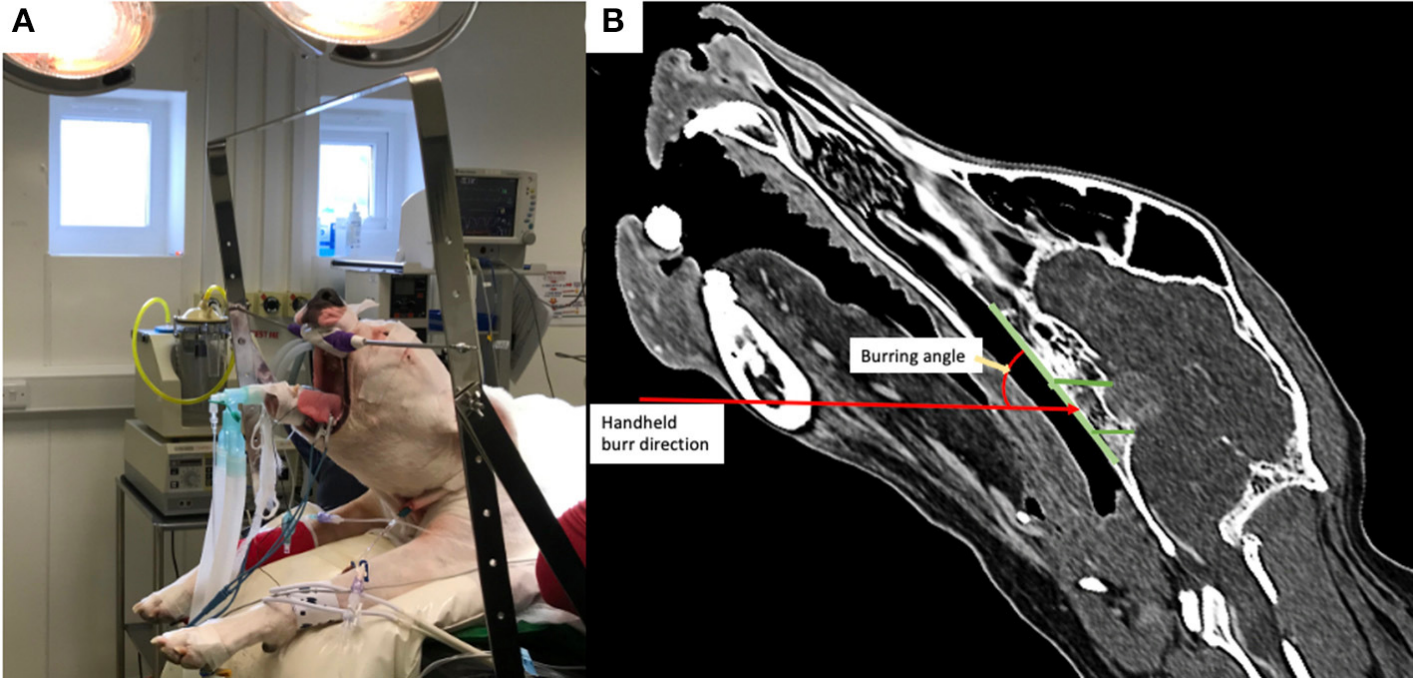
**(A)** Photograph of a dog positioned in theatre with the head attached by the canine teeth to a metallic bar; note the head is therefore tilted from horizontal; **(B)** post-operative reconstructed sagittal CT image in a bone window showing the angulation required for burring with the handheld drill into the basisphenoid bone.

All dogs were positioned in sternal recumbency with their head held by hooking the canine teeth on a bar supported by a metal frame secured to the surgical table (*n* = 4, Figure 2) or using a commercially available surgical headframe (*n* = 4; Brainsight^TM^, Rogue Research, Canada), while avoiding pressure to the jugular veins, as previously described (1, 8, 15, 16, 20, 22). The mandible was kept hanging to allow opening of the mouth during the different steps of the surgery. The cuffed armed endotracheal tube was attached to the lower jaw and retracted to the side of the mandible out of the surgical field. Following draping, a swab with a radio-opaque marker was placed in the pharynx to prevent leakage of fluids into the airways in 4 dogs. The soft palate was incised with a n°15 blade (*n* = 4) or monopolar diathermy (*n* = 4) in the midline (taking care not to section the soft palate along its entire length) following palpation of the pterygoid *hamuli* processes. Mini Gelpi retractors were placed in the soft palate wound to visualise the palatine mucosa and mucoperiosteum overlying the basisphenoid bone and these were elevated with a freer. The 3D printed guide was then inserted into the oral cavity, adjusting it to the upper arcade molars and making sure that the arm of the guide extended to make contact with the basisphenoid bone (see result section below). Once in place, the air-powered drill was brought in contact with the basisphenoid bone to burr the window depicted by the arm of the surgical guide. During that step, the surgeon needs to maintain the burr hand piece relatively horizontal in order to burr the basisphenoid bone with an angle of ~45 degrees while the head is held tilted in position (Figure 2). Once the margins of the slot were created, the 3D patient specific surgical guide was removed from the oral cavity and the burring was continued through the cancellous bone toward the inner cortical layer until the dura underneath the pituitary gland was visible. Between burring, the surgical field was flushed with sterile saline NaCl 0.9% (B|Braun, Melsungen, Germany). Haemorrhage from the cancellous bone was controlled with bone wax (Ethicon, Johnson&Johnson Medical GmbH, Germany). The dura mater was incised with a n°11 surgical blade, after which the pituitary gland / mass lesion was visible through the incision. The pituitary gland and pituitary neoplasm were slowly and carefully removed as much as possible using blunt dissection and suction. Visualisation of the third ventricle, normal brain tissue or cerebrospinal fluid leak gave evidence of debulking of the mass. The slot created in the basisphenoid bone was covered by an absorbable collagen fleece (Lyostypt, B|Braun, Spain) or a corneal disc from porcine urinary bladder (Vetrix BioSIS ECM-BioSIS Plus^+^ 15 mm Multi-layer Ocular Discs- Vetrix, West Lafayette, IN 47906 USA) at the end of the surgery. The internal and external layers of the soft palate were closed in two planes with polyglactin suture (Vicryl 2-0 or 3-0, Johnson&Johnson, Belgium).

### Pre- and Post-surgical Measurements of the Burring Site

**Figure 3:**
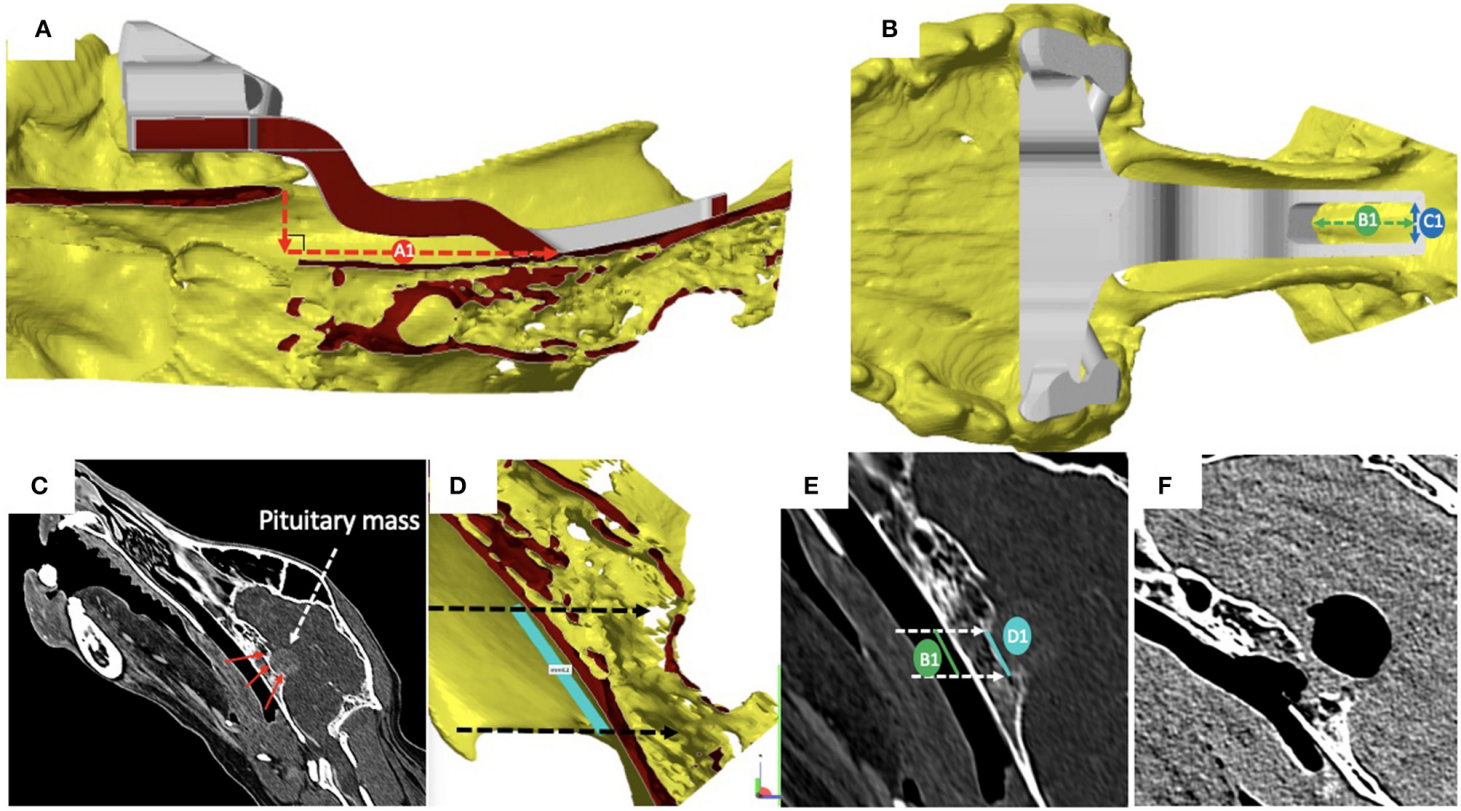
**(A)** Sagittal section of the skull visualised with the CAD software Netfabb professional (rostral is to the left in the figure); cortical surfaces appear in yellow and the 3-dimensional printed guide in place is in grey: the distance between the vomer and the rostral aspect of the burring window depicted by the guide (A1) was measured by drawing a vertical line from the tip of the vomer to basisphenoid and another line, perpendicular to the first and extending to the rostral margin of the surgical guide; **(B)** dorsal view of the skull and surgical guide in place, visualised with the CAD software: the length (B1) and the width (C1) of the burring window into the basisphenoid first cortical bone layer appear as the green and blue double head dashed arrows, respectively; **(C)** the length of bone to remove from the inner cortical layer of the basisphenoid bone (red arrows) forming the *sella turcica* was defined to match that of the outer cortical layer to burr; **(D)** light blue line chosen to be no longer than the *tuberculum sellae* and the *dorsum sellae*); **(E)** the length of inner and outer cortical layers to be removed where defined as equal; **(F)** sagittal reconstructed post-operative post-contrast CT image obtained to assess completeness of the hypophysectomy; note the signal void region suggesting presence of air.

**Figure 4:**
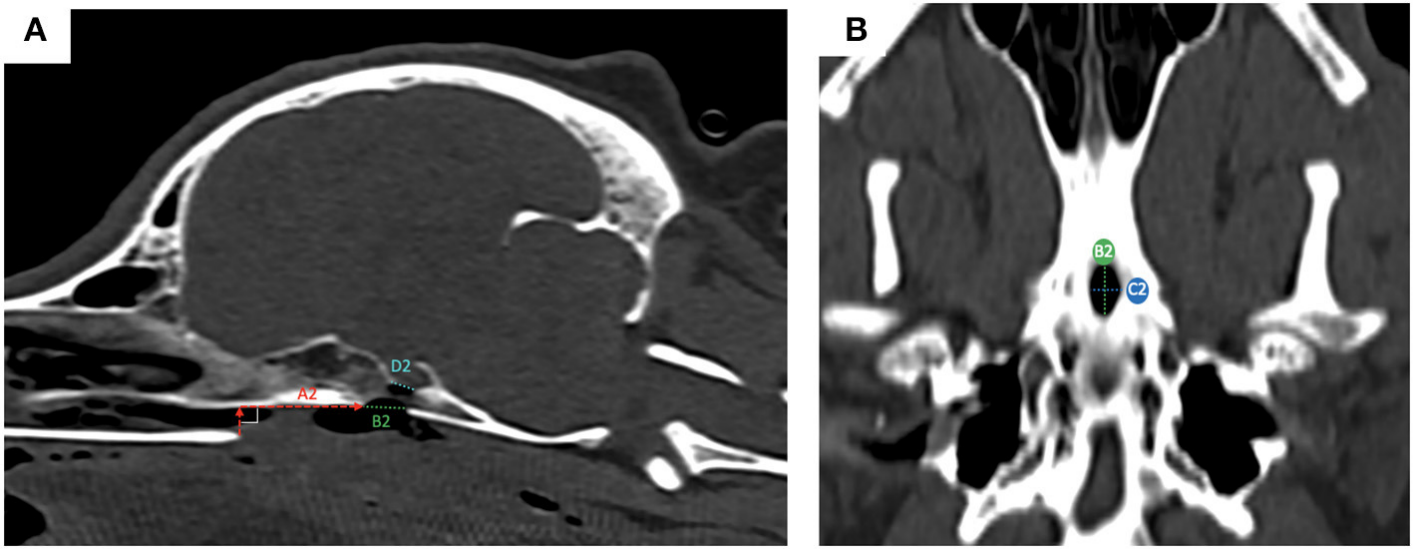
post-operative measures from reconstructed sagittal CT images in a bone window; the measured obtained were: **(A)** the distance between the vomer and the rostral aspect of the achieved window in the basisphenoid outer cortex (A2, red dashed horizontal line); (ii) the length of bone removed from the outer cortical layer (B2, green dashed line) and the length of bone remove from the inner cortical layer (D2, light blue dashed line); **(B)** ventral view of the basisphenoid bone: the width (C2, blue dashed line) of the achieved window is visible, as well as the length (B2, green dashed line).

Pre-operatively, we defined and measured (Figures 3A,B): (i) the distance between the vomer and the rostral aspect of the burring window depicted by the guide (named A1); (ii) the length (named B1) and the width (named C1) of the burring window into the basisphenoid outer cortical bone layer, as depicted by the guide in place; and (iii) the length of cortical bone removed from the second cortex forming the *sella turcica* (in a sagittal plane, named D1) and chosen to be the same as B1 (Figures 3C–E). All cases underwent a post-operative CT-scan [before and after intravenous injection of iohexol contrast at a dose of 1.7 ml/kg (Omnipaque, GE Healthcare, USA)] immediately after surgery to assess completeness of the hypophysectomy (Figure 3F). From the DICOM images, the following parameters were measured again (see Figures 4A,B): (i) the distance between the vomer and the rostral aspect of the achieved burred window in the basisphenoid outer cortex (named A2); (ii) the length (named B2) and width (named C2) of the achieved window in the basisphenoid outer cortex, and (iii) the length of cortical bone removed from the second cortex forming the *sella turcica*, named D2. Assessment of the accuracy of the burred window was evaluated by comparing the pre- and post-operative measures.

For the measurements, the median and the range are reported. Comparison of pre- and post-operative measures was done with the non-parametric Wilcoxon test for paired data with a *p* value of 0.05 considered significant and using GraphPad Prism version 9.0.0 for Windows (GraphPad Software, San Diego, California USA, www.graphpad.com).

We defined that a variation from baseline to post-operative measures of a magnitude of <10% would be acceptable and safe based on the distance left between the margins of the burred window and the cavernous sinuses and arterial circle of Willis measured on the post-contrast CT images.

## Results

### Case Signalment

Eight dogs with pituitary-dependent hyperadrenocorticism underwent transsphenoidal hypophysectomy with the help of a 3D printed surgical guide. Five patients were male (4 neutered and 1 entire) and 3 were neutered females. The age at the time of surgery ranged from 5.8 to 12.4 years (median age was 8 years old). The body weight ranged from 8.65 to 34.3 kg (median body weight was 29 kg). The breeds were: Labrador retriever (*n* = 3), Greyhound (*n* = 1), Border terrier (*n* = 1), Boxer (*n* = 1), English springer spaniel (*n* = 1), and Bichon frise (*n* = 1). The two cadavers were a Boxer and a Greyhound. The median P/B ratio was 0.37 and ranged from 0.29 to 0.85 for the eight clinical cases (Table 1); therefore, two cases were classified as non-enlarged adenoma and six were classified as enlarged adenoma. There was no involvement of the arterial circle of Willis or cavernous sinus in any of the cases, except in case 4.

**Table 1:** Signalment, pituitary height/brain area ratio (P/B ratio), and clinical outcome in the eight dogs in which transsphenoidal hypophysectomy was performed with the use of the 3D-patient-specific surgical guide.

**Cases**	**Breed**	**Age (years)**	**Sex**	**Body Weight (kg)**	**P/B ratio (mm^**−1**^)**	**Outcome; survival to date**	**Histopathology**
Case 1	Greyhound	7.3	FN	32	0.80	Clinical remission (absent hyperadrenocorticism); 34 months	Chromophobic enlarged adenoma
Case 2	Labrador retriever	5.8	MN	34.3	0.36	Clinical remission (absent hyperadrenocorticism); 29 months	Sinusoidal chromophobe adenoma
Case 3	Border terrier	12.4	MN	9.7	0.85	Mild persistent right-sided head tilt; persistent hyperadrenocorticism; 20 months	Sample of non-diagnostic quality
Case 4	Boxer	8	ME	26.3	0.78	Euthanasia 7 months post-operatively	Sinusoidal adenoma
Case 5	Labrador retriever	9	MN	29	0.70	Euthanasia 24 h post-operatively	Sample of non-diagnostic quality
Case 6	Labrador retriever	8	MN	30.6	0.30	Clinical remission (absent hyperadrenocorticism); 8 months	Sinusoidal adenoma
Case 7	English springer spaniel	11	FN	20.7	0.38	Clinical remission (absent hyperadrenocorticism); 8 months	Acidophil adenoma
Case 8	Bichon frise	9	FN	8.65	0.29	Clinical remission (absent hyperadrenocorticism); 6 months	Sinusoidal chromophobe adenoma

*FN, female neutered; MN, male neutered; ME, male entire*.

### Guide Design and Placement in Surgery

**Figure 5:**
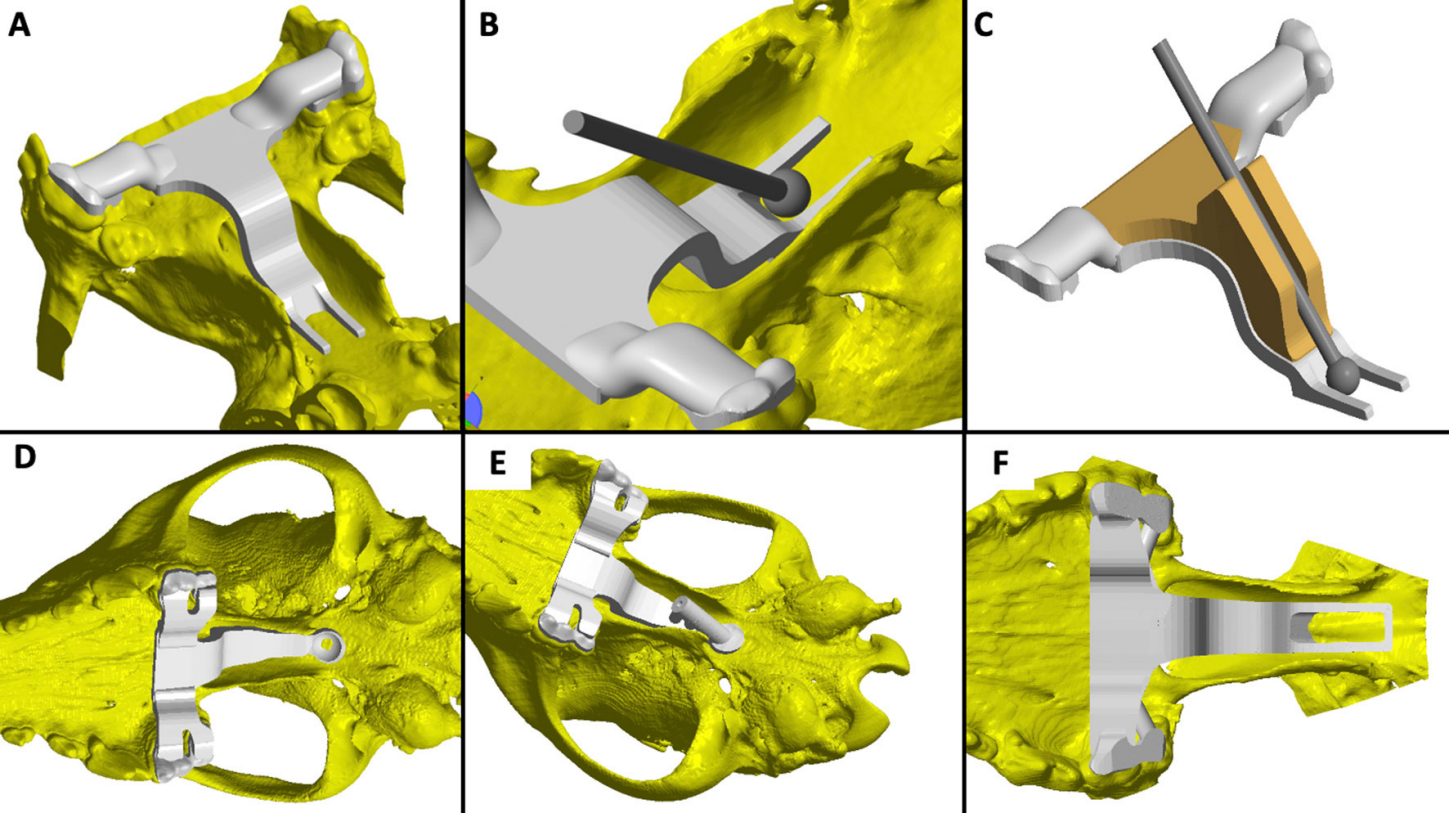
Examples of the various iterations of the guide discussed by the authors: **(A)** the caudal aspect of the guide window left open; **(B)** placement of a slight slope at the rostral edge of the burring window to aid with burring trajectory; **(C)** use of a semi-opened channel to guide the bur; **(D)** guide offering solely a pilot hole position; **(E)** full channel to guide a pin and place a pilot hole; **(F)** final and preferred design.

**Figure 6:**
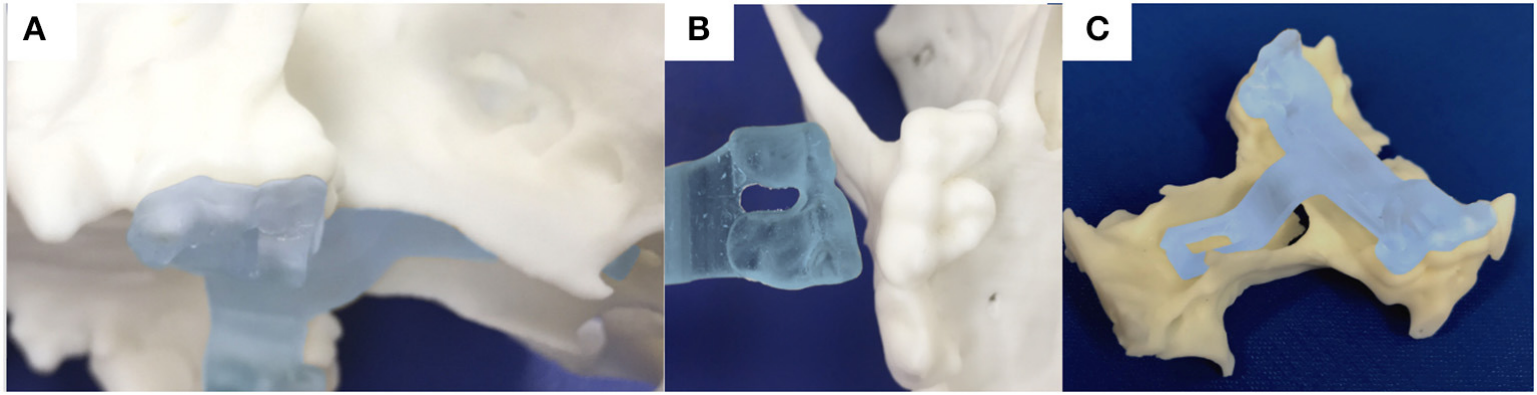
photograph of the 3-dimensional printed skull showing the surface of the molar teeth on the left side in sagittal **(A)** and ventral **(B)** views; **(C)** the printed guide can lock on the molar teeth and contains an arm extending toward the basisphenoid bone with a shape following the anatomical contour of the hard palate, while avoiding the vomer bone and eventually reaching the outer cortex of the basisphenoid bone.

**Figure 7:**
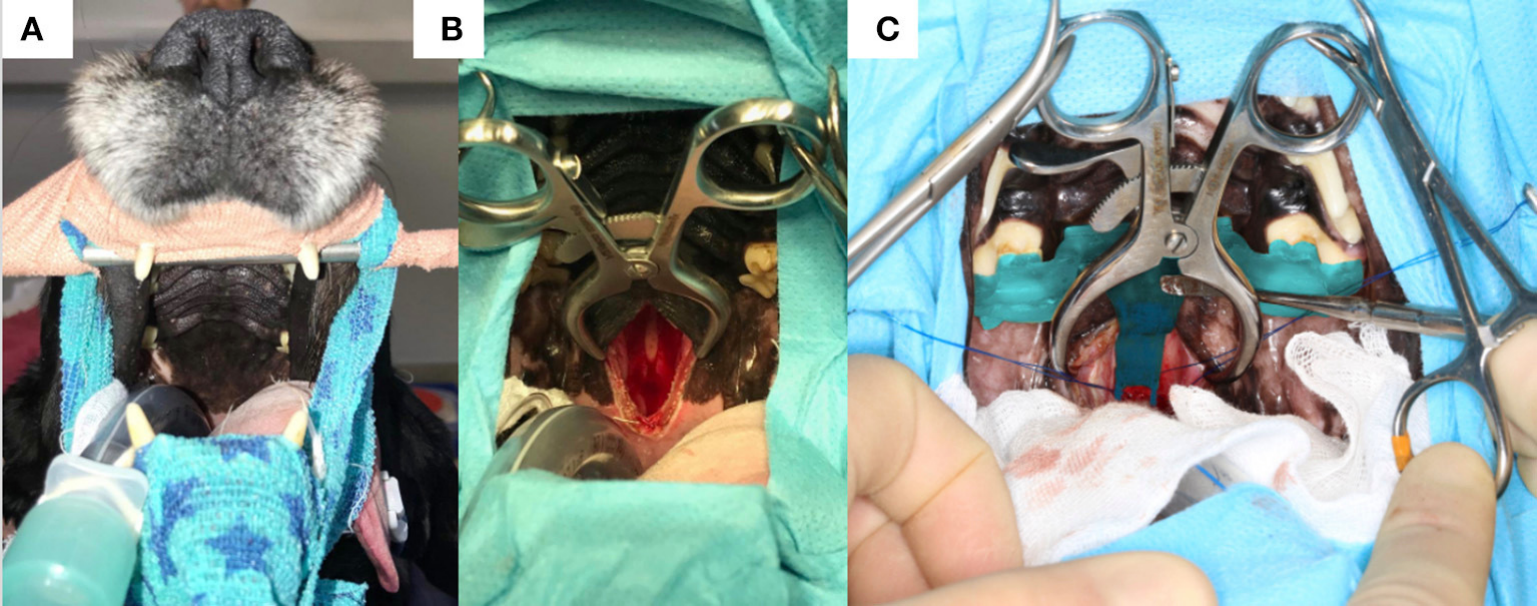
**(A)** Photograph of the head of one of the dogs before surgery with the canines hooked on a bar attached to a frame and the surgical table; **(B)** incomplete incision in the soft palate and palatine mucosa exposing the basisphenoid bone; **(C)** 3-dimensional printed surgical guide *in situ* held by an assistant with a surgical instrument; note the foot of the guide extending toward the planned burring window; the Gelpi retractors also help to maintain the guide in place.

Following dissection of the cadavers, CT, and study of the 3D printed skulls in the CAD software (Figures 1A,B), multiple ideas were discussed between the authors in terms of what would be the most convenient design and location for the guide within the oral cavity (Figure 5). It appeared that locking the guide onto the upper arcade molars was the best option because the ventral aspect of the molars offers a well-contoured surface for purchase of the guide and is unique to each dog (Figure 6). This would ensure stability of the surgical guide and the molar teeth are easily reachable with the oral cavity open whilst doing open mouth surgery. Even if an animal was lacking one of the molars, the guide could be designed to be fitted onto a more rostral tooth. The abaxial aspects of the guide therefore comprised a contact of footprint which was an inverted representation of the maxillary molars (Figure 5B). We then designed an arm extending toward the basisphenoid bone with a shape following the anatomical contour of the hard palate, while avoiding the vomer bone and eventually reaching the region of the basisphenoid bone we wished to burr away (Figure 6C). Several designs were considered for the guide “foot” (Figure 5), including a U-shape, a round shape, a tunnel shape that could accommodate a burr or a pin and/or a rectangular shape. Eventually, the rectangular shape was retained, as it was the one restricting the least the surgeon's view once the guide was in place. The base of this foot was an inverted representation of the cortical contours of the basisphenoid bone at that point, thus providing a third point of contact to optimise guide fit and stability. The length of the rectangular window was designed to match the distance between the *tuberculum sellae* and the *dorsum sellae* (Figures 3C–E) and the width was dictated by the width of the *sella turcica* and need to avoid vascular structures. The angle of the rostral wall of the guide window was designed to match the necessary angle for the bone tunnel to emerge at the rostral aspect of the *sella turcica* and could be used as guide by the surgeon. We initially planned to use surgical glue to hold the guide in position, but this was discarded after the experience of the first surgery because it was felt to be convenient to be able to move the guide in and out of the mouth during the approach. Indeed, the guide was designed to indicate the burring site in the outer cortical layer of the basisphenoid bone and then removed to continue the approach through the basisphenoid bone. It was concluded that the guide could be held in place by a surgical assistant by applying gentle pressure toward the molars until the burring window was completed (Figure 7 and Supplementary Video 1); Gelpi retractors placed to maintain the soft palate wound open also helped to maintain the guide. The variation between A1-A2, B1-B2, C1-C2, and D1-D2 was 0.03, 0.10, 0.00, and 0.30 cm, respectively, for the first cadaver and 0.00, 0.30, 0.10, 0.30 cm, respectively, for the second cadaver.

Trial in more cadavers was not considered necessary, since in case the guide would fail, the surgery could still be performed as previously described (15, 20).

When used *in vivo*, the guide was successfully held in place by the assistant. The surgeons observed that depending on the thickness of the soft palate and pre-planned contour of the guide, further dissection of the rostral aspect of the soft palate and even the hard palate in case 4 (a Boxer where the view was restricted due the brachycephalic shape of the head), was required for a perfect fit. This also facilitated the guide's stability.

### Pre- and Post-operative Measures in Operated Cases

For the pre-operative measures, we found that A1 had a median of 2.22 cm and ranged from 1.30 to 3.05 cm; B1 had a median of 1.25 cm and ranged from 0.7 to 1.45 cm, C1 had a median of 0.5 cm and ranged from 0.40 to 0.60 cm and D1 had a median of 1.25 cm and ranged from 0.70 to 1.45 cm. For the post-operative measures, we found that A2 had a median of 2.2 cm and ranged from 1.10 to 3.06 cm, B2 had a median of 1.17 cm and ranged from 0.60 to 1.60 cm, C2 had a median of 0.55 cm and ranged from 0.40 to 0.70 cm and D2 had a median of 1.17 cm and ranged 0.7 to 1.35 cm. We entered the *sella turcica* in all cases and did not breach into the *tuberculum sellae* rostrally or *dorsum sellae* caudally. For A2 and A1, the median of differences was −0.075 mm (ranging from 0.00 to 0.20 cm); for B2 and B1 the median of differences was −0.05 mm (ranging from 0.00 to 0.30 cm); for C2 and C1 the median of differences was −0.015 mm (ranging from 0.00 to 0.20 cm); and for D1 and D2 the median of differences was −0.1 mm (ranging from 0.00 to 0.20 cm) (Table 2). The median of differences for the 4 parameters studied were all <10% of variation from baseline. There was no significant difference between the pre- and post-operative measures: (i) A1 vs. A2: *p* = 0.1875, *W* = −16; (ii) B1 vs. B2: *p* = 0.2031, *W* = −16; (iii) C1 vs. C2: *p* = 0.6250, W = 6; (iv) D1 vs. D2: *p* = 0.2344, *W* = −15 (Wilcoxon test).

**Table 2:** Pre- (A1, B1, C1, D1) and post-operative (A2, B2, C2, D2) measures obtained from eight cases undergoing hypophysectomy with the use of a 3-dimensional printed guide.

**Case number**	**Parameter**	**Pre-operative measures (A1; B1; C1; D1) (cm)**	**Post-operative numbers (A2; B2; C2; D2) (cm)**	**Difference between A1-A2, B1-B2, C1-C2 and D1-D2 (cm)**	**Variation from baseline (%)**
	A	2.10	2.20	0.10	4.7
1	B	1.30	1.25	0.05	3.8
	C	0.50	0.60	0.10	20
	D	1.30	1.10	0.20	15
	A	3.05	3.06	0.01	0.3
2	B	1.45	1.43	0.22	1.3
	C	0.60	0.70	0.10	16.6
	D	1.45	1.30	0.15	10.3
	A	1.30	1.10	0.20	15.3
3	B	1.40	1.60	0.20	14.3
	C	0.40	0.60	0.20	50
	D	1.40	1.30	0.10	7.1
	A	2.35	2.20	0.15	6.3
4	B	1.20	1.10	0.10	8.3
	C	0.50	0.41	0.09	18
	D	1.20	1.15	0.05	4.1
	A	2.60	2.70	0.10	3.8
5	B	1.00	1.00	0.00	0
	C	0.43	0.40	0.03	6.9
	D	1.00	1.20	0.20	20
	A	2.40	2.40	0.00	0
6	B	1.20	0.90	0.30	25
	C	0.46	0.43	0.03	6.5
	D	1.20	1.10	0.10	8.3
	A	1.90	1.70	0.20	10.5
7	B	1.45	1.40	0.05	3.4
	C	0.60	0.60	0.00	0
	D	1.45	1.35	0.10	6.8
	A	1.8	1.63	0.17	9.4
8	B	0.7	0.6	0.10	14.2
	C	0.6	0.5	0.10	16.6
	D	0.7	0.7	0.00	0

### Clinical Outcome

For all 10 dogs, including both cadavers, entry into the *sella turcica* was achieved allowing removal of the pituitary gland and all (*n* = 6) or some of the mass lesion (*n* = 2).

None of the cases had intra-operative complications or died during surgery. Case 1 suffered from a cerebrovascular accident 5 days post- operatively and 5 months later was in clinical remission and has remained so since then. At 4 weeks after surgery, ACTH level was < 5pg/ml in this case, confirming the absence of hypercortisolism. Case 2 also had an ACTH measurement < 5 pg/ml 4 weeks after surgery, which confirmed absence of hypercortisolism. This case remains in remission. Case 3 developed post-operative right-sided vestibular signs which gradually improved. In this case, complete excision of the mass was not possible, therefore this patient remains with clinical signs of hyperadrenocorticism (ACTH 4 weeks after surgery was 64 pg/ml) and with a subtle head tilt to the right. Case 4 developed severe aspiration pneumonia 72 h after surgery which resolved with medical treatment. However, 7 months following surgery the patient had severe haemorrhagic diarrhoea and vomiting which led to decreased mentation and generalised weakness and the owner elected euthanasia. Case 5 showed loss of vision and pupillary light reflexes post-operatively, which was suspected to be secondary to haemorrhage and the owner elected euthanasia. Cases 6 and 7 are in clinical remission and both had ACTH levels 4 weeks after surgery <5 pg/ml and are not showing any sign of Cushing's disease. Case 8 had surgery in October 2021 and is neurologically normal 6 months later.

The follow-up in this case series ranges from 1 day to 34 months and the survival at 6 months is 75% (Table 1).

On histopathology, 5 dogs were diagnosed with a pituitary chromophobe sinusoidal adenoma, 1 dog with a pituitary acidophil adenoma and 2 dogs (cases 3 and 5) were diagnosed with pituitary neoplasia, however the sample was insufficient to adequately differentiate between benign or malignant pituitary neoplasia.

## Discussion

This study shows that the use of a 3D patient specific surgical guide is a possible method to localise the burring site along the outer cortical layer of the basisphenoid bone during hypophysectomy. The described technique was accurate to a tenth of a millimetre, which we consider satisfactory. This is similar to the accuracy desired for some brain surgeries in humans and reached with the use of 3D printed surgical guides, for example for implantation of deep brain stimulation electrodes (23).

A recent study from colleagues in Korea, using experimental dogs under 8.5 kg, performed coincidentally and in parallel to ours, also reports that a 3D surgical guide for transsphenoidal hypophysectomy provides an accurate reading of the entry point into the outer cortical layer of the basisphenoid bone (24). These authors have not tested their device in companion dogs affected by pituitary dependent-hyperadrenocorticism (24). They focus on the accuracy of a single pilot hole indicating the center of the *sella turcica* that is later extended in a rectangular shape in cranio-caudal and latero-lateral directions (24). The pre-operative planned measures were 6 mm in width and 8 mm in length, and the post-operative measures had a median of 5.17 mm width and 7.51 mm length, therefore similar to the precision we achieved (24).

It is important to recognise that the device we propose only gives the location of the burring site at the level of the outer basisphenoid cortical layer. Whilst it can provide the surgeon with a burring trajectory or angle through the basisphenoid bone to reach the inner cortical layer and therefore the *sella turcica*, indicated by the rostral wall of the guide window, it is a small surface that is difficult to follow and may not be reliable. In addition, the head position of the dog, tilted ~45 degrees from horizontal, renders burring in the basisphenoid bone challenging. It is possible that other intraoperative tools such as goniometers could be thought to ascertain the optimal angle of approach of the *sella turcica* based on preoperative CT imaging. It might be that modification of the guide, to visualise the burring angle more clearly is possible too and something we are working on. Eventually, we did enter the *sella turcica* in all cases with a precision of a tenth of a centimetre. This seems small, however, the space we wished to create in the inner layer of the basisphenoid bone ranged from 0.7 to 1.35 cm and therefore, in some cases, a 0.1 mm variation would be above the 10% we considered acceptable. This is an imprecision we need to resolve through further research. Other clinicians using neuronavigation systems have also reported errors in localisation both in dogs and in humans, due to the variation in size, shape and thickness of the skull conformation, making the application of surgical systems challenging (25, 26). This also serves to highlight the importance of good surgical technique and clear understanding of the skull base anatomy required to perform this procedure. The impact of the use of this device on the outcome of the dogs was not a goal in this study and should be investigated as we continue to use this system. The post-operative mortality we recorded within 4 weeks was 12.5% (case 5, with a P/B ratio = 0.7 mm^−1^, was euthanised 24 h after surgery). Eighty seven percent of the dogs (7 dogs) survived more than 4 weeks and of those cases, all but one (case 4), are alive at the time of writing. These survivals are similar to those previously published (4, 8, 9, 18). Further, in our cases (except case 4), there was no involvement of the arterial circle of Willis or cavernous sinus, similar to grade 2 and 3A cases presented by Sato et al. (18) and for which a good clinical outcome is expected (18).

Historically, hypophysectomy *via* an intracranial transtemporal route was used in experimental dogs but was later replaced by the transsphenoidal route (3). Reported transsphenoidal techniques in experimental “normal” dogs include: (i) the placement of three small self-threading screws in the basisphenoid bone used as radiographic markers and followed by cranial sinus venogram outlining the *sella turcica* (16); (ii) a ventral paramedian approach using CT images obtained with the tip of a radiopaque feeding tube placed in the nasopharynx near the *hamuli* processes (10); (iii) the use of a stereotactic neuro-navigation system (27); and (iv) the use of a 3D surgical guide using CT and MRI images for the design and planning of the device (24). In practise, the reported transsphenoidal techniques in pet dogs with pituitary dependent hyperadrenocorticism include: (i) using anatomical landmarks such as the *hamuli* processes and emissary vein; and (ii) placement of pilot holes in the outer cortical layer of the basisphenoid bone followed by CT imaging (4, 12, 14, 20). These procedures can be invasive, imply peri-surgical imaging and therefore the need to take the animal out of theatre to CT and back to theatre, therefore increasing surgical time. Even then, the gain in precision for the surgeon to burr the basisphenoid bone hole accurately remains unknown. For the technique used by Owen et al. (9) the neuro-navigation system involves an expensive investment that is unlikely to be acceptable for medium-sized referral hospitals and comes with the need to place a frame around the head in the dog, which requires creating skin wounds to place the fixing pins (27). In comparison, the device we are proposing has a low profile (i.e., is thin enough) that leaves the burring site visible to the surgeon, is non-invasive and is rapidly deployable in surgery. It can be placed for practise, removed, and replaced in the mouth at will. It is also individually customised to the patient taking into account anatomical variations. We believe that this technique adds a useful option for neurosurgeons performing this procedure, thanks to the advances offered by 3D printing and the direct use of CT DICOM images within CAD software. Further, this surgical guide could be designed to fit varying skull sizes and shapes—our smallest patient was 9.7 kg dog and the largest patient was 34.3 kg—and if the patient is lacking any of the molar teeth, the guide could be designed using other teeth. The present study did not compare surgery duration with other surgical techniques due to the small number of cases available. However, we feel that the device, regardless of the gain in surgical time, can offer additional comfort to the surgeon and increase accuracy in locating the entry point in the basisphenoid bone, thereby increasing the surgeon's confidence.

Another limitation of this surgical guide is the cost, which, in the UK veterinary market, adds ~10% to the total cost of the procedure. On the other hand, the use of the guide might remove the need for peri-operative CT or other neuronavigation techniques.

Hypophysectomy is typically an elective procedure to treat a chronic condition hence there is no need for a fast turn-around. The time needed to design and produce a guide can be as low as 3 days; it took on average 1 week to do in the cases presented.

It is known that pituitary surgery requires a learning curve for the surgeon to become confident in performing the approach. In 1999, Meij et al. clearly recognised this issue and reported five deaths related to the procedure in their first series of 26 cases, whereas they faced no death in their second series of 26 cases (1). Mamelak et al. also reported this in 26 operated dogs where the mortality rate was 50% in the first 10 cases and 0% in the next 16 dogs (14). Without replacing the need for training for this surgery, we propose that the extra help provided by our device in locating the entry point in the basisphenoid could improve the learning curve, although this is something we would need to study in another case series. A multicenter study would allow to collect a greater number of cases to better assess this theory. The use of 3D printing technology to train doctors to perform trans-nasal sphenoid endoscopy and hypophysectomy in people has been reported too (28). In that instance, 3D printing is used to model the hard and soft tissues of the skull region of interest and the neurosurgeon can then navigate their endoscope in the replica of the patient's head to gain familiarity with the anatomy.

Anecdotally, we have used the surgical guide for one feline acromegalic patient undergoing hypophysectomy, a treatment previously described (2, 3, 6, 22). We obtained the same precision as in the dogs of this study to locate the entry point in the basisphenoid bone. That cat has remained in clinical remission at the time of writing (16 months) with normalised insulin-like growth factor. To form a homogenous case series, we have decided to exclude this cat from the study since it would not add any meaningful data. But this suggests that the surgical device could be used in cats and is something we wish to test.

In conclusion, this surgical device provides an accurate method to localise the burring site in the outer layer of the basisphenoid bone when performing transsphenoidal hypophysectomy in dogs. It is versatile, non-invasive, and easy to apply. Whilst it does not replace the need for a thorough understanding of the surgical technique and relevant anatomy, and the need for training, it may prove useful as a teaching tool for veterinary specialist neurosurgeons wishing to learn to perform a hypophysectomy.

## Data Availability Statement

The original contributions presented in the study are included in the article/Supplementary Material, further inquiries can be directed to the corresponding authors.

## Ethics Statement

Ethical review and approval was not required for the animal study because the authors perform hypophysectomy routinely in their clinics as a treatment for Cushing's disease. The dogs presented in the manuscript have undergone hypophysectomy as part of their treatment plan and for their direct benefit. The interventions therefore were done under the Veterinary Surgeons Act (VSA) 1966. The guides used in the dog's mouth were a visual aid, as much as loupes we use for this surgery or other surgical tools, and did not require any additional intervention; it does not involve breaching the skin, mucosa or any invasive action on the dog. We have explained the surgery to all clients and collected informed consent from the owners of these cases and permission to use medical data. Written informed consent was obtained from the owners for the participation of their animals in this study.

## Author Contributions

BO, NG, and LE designed the guide. NG designed the study. BO ran the CAD software and 3D printing to manufacture the guide. JF, JM, and NG contributed to the article by using the device in surgery. LE, JF, HV, GN, JM, NG, and DR were involved in the clinical management of the cases. LE and NG wrote the manuscript. DR contributed to the article by performing the diagnostic investigations and advising on CT technique for later 3D printing. All authors proof read and helped correcting the manuscript. All authors contributed to the article and approved the submitted version.

## Funding

The publication fees was funded by CVS Group PLC Company.

## Conflict of Interest

BO is the manufacturer of the surgical device through the company Vet3D and NG undertakes paid consultancy work for Vet3D in relation to the guide system. The remaining authors declare that the research was conducted in the absence of any commercial or financial relationships that could be construed as a potential conflict of interest.

## Publisher's Note

All claims expressed in this article are solely those of the authors and do not necessarily represent those of their affiliated organizations, or those of the publisher, the editors and the reviewers. Any product that may be evaluated in this article, or claim that may be made by its manufacturer, is not guaranteed or endorsed by the publisher.

## Abbreviations

P/B: pituitary height/brain area.
